# Electrochemical scaffold generates localized, low concentration of hydrogen peroxide that inhibits bacterial pathogens and biofilms

**DOI:** 10.1038/srep14908

**Published:** 2015-10-14

**Authors:** Sujala T. Sultana, Erhan Atci, Jerome T. Babauta, Azeza Mohamed Falghoush, Kevin R. Snekvik, Douglas R. Call, Haluk Beyenal

**Affiliations:** 1 School of Chemical Engineering & Bioengineering, Washington State University, Pullman 99163, WA; 2 Department of Veterinary Microbiology and Pathology, Washington State University, Pullman 99163, WA; 3 Washington Animal Disease Diagnostic Laboratory, Washington State University, Pullman 99163, WA; 4 Paul G. Allen School for Global Animal Health, Washington State University, Pullman 99163, WA

## Abstract

We hypothesized that low concentrations of H_2_O_2_ could be generated through the electrochemical conversion of oxygen by applying an electric potential to a conductive scaffold and produce a low, but constant, concentration of H_2_O_2_ that would be sufficient to destroy biofilms. To test our hypothesis we used a multidrug-resistant *Acinetobacter baumannii* strain, because this species is often implicated in difficult-to-treat biofilm infections. We used conductive carbon fabric as the scaffold material (“e-scaffold”). *In vitro* experiments demonstrated the production of a maximum constant concentration of ~25 μM H_2_O_2_ near the e-scaffold surface. An e-scaffold was overlaid onto an existing *A. baumannii* biofilm, and within 24 h there was a ~4-log reduction in viable bacteria with an ~80% decrease in biofilm surface coverage. A similar procedure was used to overlay an e-scaffold onto an existing *A. baumannii* biofilm that was grown on a porcine explant. After 24 h, there was a ~3-log reduction in viable bacteria from the infected porcine explants with no observable damage to the underlying mammalian tissue based on a viability assay and histology. This research establishes a novel foundation for an alternative antibiotic-free wound dressing to eliminate biofilms.

Multidrug-resistant *Acinetobacter baumannii* is an example of an organism that is increasingly linked to nosocomial infections on wound surfaces^1^. Biofilm removal from such wounds is paramount because otherwise biofilm delays the healing process and results in a chronic wound infection. Because biofilm communities are at least partially protected from antibiotics^2^,^3^,^4^,^5^,^6^, complete eradication can be challenging. As an alternative, several antimicrobial scaffolds have been developed to dress wounds and remove biofilm infections. These scaffolds are usually “loaded” with a high concentration of an antibacterial compound [silver, zinc, iodine or honey^7^,^8^,^9^,^10^,^11^]. From a kinetics perspective, this means that the scaffold loses potency over time as the concentration gradient diminishes^12^. No existing scaffolds are capable of continuous delivery of an antimicrobial agent at a constant concentration for any significant length of time.

Electrical stimulation (ES) was originally advocated over a century ago for wound treatment^13^,^14^,^15^,^16^. ES can eliminate biofilms from infected wound surfaces and thus enhance wound healing. Importantly, however, ES did not receive significant attention in the past because we lacked an understanding of the antibacterial mechanisms involved and consequently a means to standardize ES applications^13^,^14^,^15^,^16^,^17^,^18^. Recent advances in the use of electrical phenomena in biological systems have triggered renewed interest in ES as an alternative therapy for biofilm-infected wounds^18^. The application of ES via direct current (DC) has been the most effective method for wound healing based on the measured wound healing rate in several *in vitro, in vivo* and animal model studies^13^,^14^,^16^,^17^,^19^. Despite the apparent effectiveness of DC, the mechanism by which ES improves wound healing remains unknown^18^. This is best illustrated by the examples summarized in Table 1, which report contradictory conclusions for DC applications.

Previous studies employed a range of DC voltage, current setting, polarity of the electrode placed on a wound infection, length of application time and other variables (Table 1). As a consequence, it is difficult to draw conclusions about the general effectiveness of DC-based ES as a therapeutic treatment^18^. For instance, an electric current of 32 μA/cm^2^ applied through a copper mesh electrode with negative polarity for 2 h, three times a day, eliminated *P. aeruginosa* from infected skin ulcers^16^. In contrast, the application of a 52-μA/cm^2^ electric current through the same electrode material with negative polarity required 72 h of continuous application to eliminate *P. aeruginosa* from an infected wound model successfully^15^. Most investigators speculate that electrical current is responsible for antibacterial effects, but no mechanisms have been confirmed^15^,^16^. Others have applied DC voltage (3.5 V) to inhibit *P. aeruginosa* on an electrode surface and speculated that toxic compounds are responsible^20^, but this mechanism has not been confirmed either^13^,^14^,^20^. Thus, despite numerous hypotheses regarding the mechanism of action of ES, there is no unifying theory on which to standardize treatments to eliminate biofilm from wound infections or standardize investigations^18^. This lack of understanding likely derives, in part, from too little emphasis being placed on the role of electrochemical processes occurring at an electrode surface applied to a wound.

ES uses two inert electrodes to control and drive electrical current and control biofilm^21^,^22^,^23^,^24^. Until recently, however, the community has lacked the tools and methods to investigate the micro-environmental changes that are caused by electrochemical reactions^22^. Recently, our research group reported that continuous (40 h) electrochemical generation of low concentrations of H_2_O_2_ was detected near a stainless steel electrode with negative polarity and that the H_2_O_2_ appeared to delay biofilm development^22^. The electrochemical formation of H_2_O_2_ results from the partial reduction of dissolved oxygen in an aqueous solution on an electrode as per equation (1)^22^,^25^.


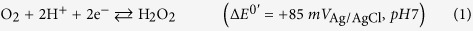


The reduction potential of H_2_O_2_ is +85 mV_Ag/AgCl_, but because of its high activation overpotential, H_2_O_2_ production usually requires a negative polarization potential^26^. When an electrode in a wound environment is polarized below +85 mV_Ag/AgCl_, oxygen will be reduced to generate H_2_O_2_, which can prevent/delay biofilm growth^22^. Depending on the concentration, the electrochemical generation of H_2_O_2_ should be compatible with wound healing because a low concentration of H_2_O_2_ is normally produced in wounds as a cellular inflammatory response and H_2_O_2_ is required for healing^27^, probably through the stimulation of keratinocyte differentiation^28^,^29^. Clearly, only a low concentration of H_2_O_2_ can be tolerated so as to avoid oxidative damage to tissue^30^,^31^. Also, such electrochemical generation of H_2_O_2_ should be continuous over time. Thus, we hypothesized that an electrochemical scaffold capable of continuous controlled delivery of a low concentration of H_2_O_2_ can function as an efficient antibiotic-free wound dressing to destroy biofilms.

Our goals were to (1) develop an electrochemical scaffold (“e-scaffold”) that would reduce dissolved oxygen to H_2_O_2_, (2) test its biocidal efficacy at eliminating *A. baumannii* biofilms grown *in vitro* and on porcine explant models, and (3) use a porcine explant model to determine whether the e-scaffold damages underlying tissue.

## Results and Discussion

As an e-scaffold material, we chose a carbon-based conductive fabric because of its biological compatibility, flexibility and wide use as both electrode and wound dressing material^32^,^33^,^34^. We standardized the operating electric potential for maximum sustained H_2_O_2_ concentration generated near the e-scaffold surface, based on microelectrode measurements^22^. Polarized e-scaffold was overlaid onto an existing *A. baumannii* biofilm for 24 h. Afterward, changes in biofilm surface coverage were quantified from biofilm images^6^,^35^ and the colony-forming units were counted. To verify that the electrochemical generation of H_2_O_2_ is the dominant mechanism for biofilm elimination, we neutralized the mechanism *in situ* by externally adding catalase to decompose the H_2_O_2_ generated by an e-scaffold in an identical *A. baumannii* biofilm. This allowed us to determine whether an e-scaffold can operate without H_2_O_2_ production. Similarly, in a separate experiment, we added H_2_O_2_ to give concentrations as experimentally observed to be generated by the e-scaffold to confirm that H_2_O_2_ is required to damage *A. baumannii* biofilms. The e-scaffold was further tested against infected porcine explants.

### H_2_O_2_ is generated at the e-scaffold surface

**Figure 1:**
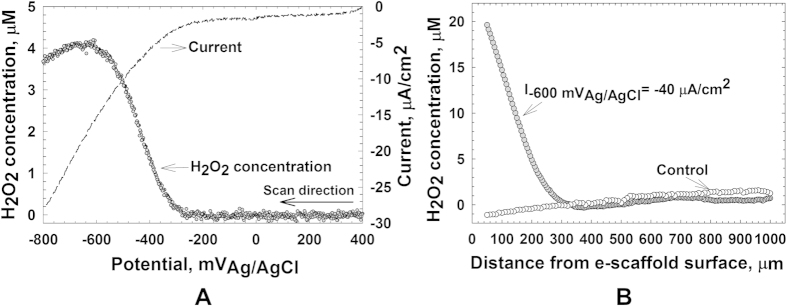
(**A**) Plot of the H_2_O_2_ concentration 50 μm from the polarized e-scaffold surface at potentials ranging from +400 mV_Ag/AgCl_ to −800 mV_Ag/AgCl_. The dashed line is the current derived from linear sweep voltammetry spectra of the e-scaffold at a scan rate of 10 mV/s. (**B**). H_2_O_2_ depth profiles for both non-polarized (control) and polarized e-scaffold surfaces. The x-axis represents the distance of the microelectrode tip from the e-scaffold surface towards the bulk, with “0” being the surface of the e-scaffold.

H_2_O_2_ becomes detectable near the e-scaffold surface when it is polarized at −300 mV_Ag/AgCl_ (Fig. 1A). This observation is consistent with the two-electron pathway (equation 1) previously described for oxygen reduction at carbon-based electrodes^32^. A maximum H_2_O_2_ concentration was detected at −600 mV_Ag/AgCl_. Consequently, for all subsequent experiments the e-scaffolds were polarized at −600 mV_Ag/AgCl_. Depth profile measurements demonstrated that the H_2_O_2_ concentration was ~25 μM at the e-scaffold surface but declined to almost zero at 300 μm from the e-scaffold surface (Fig. 1B). Such low concentrations of H_2_O_2_ are sufficient to promote wound healing by eliminating biofilm without damaging mammalian tissue^22^,^30^,^31^. As expected, the non-polarized e-scaffolds produced no detectable H_2_O_2_ (Fig. 1B). We also quantified pH profiles but observed no change in pH near the electrode surface^32^. This demonstrates that pH effects are unlikely to contribute to the e-scaffold antibacterial mechanism as had been suggested by previous papers^24^,^36^.

### *In vitro* biofilm control by e-scaffold

**Figure 2:**
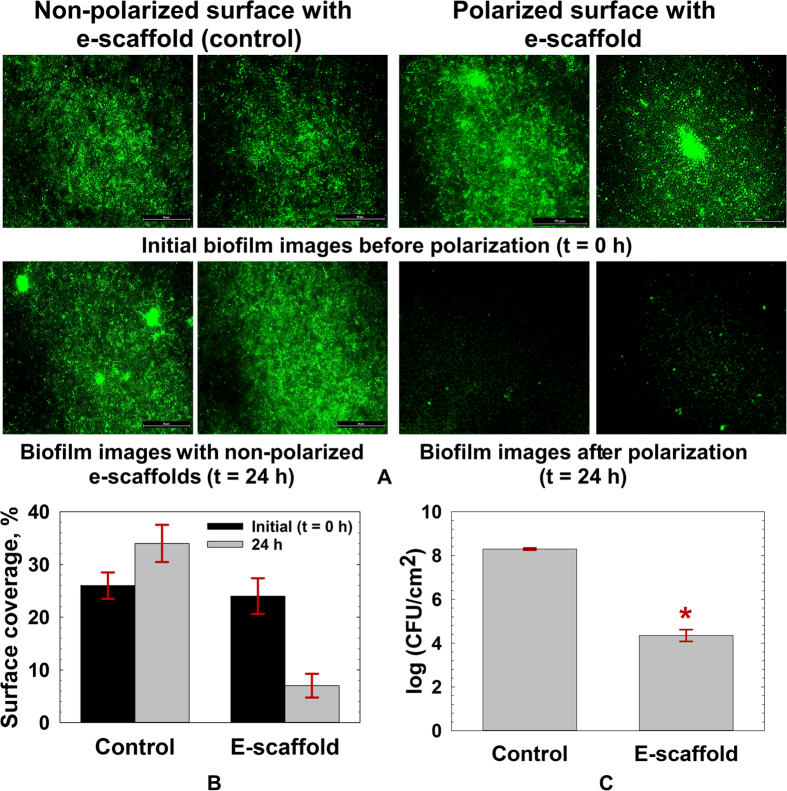
(**A**) *A. baumannii* biofilm grown *in vitro* for 1 day [initial (t = 0 h)] and after the application of e-scaffolds (24 h). The controls consisted of biofilms with non-polarized e-scaffolds. Two example images are presented for each condition; scale bar = 50 μm and magnification = 40 × magnification for all of the images. (**B**) Surface coverage for control and polarized e-scaffold treatment. The data represent means from 10 images taken for each of three independent biological replicates. The error bars represent the standard errors of the means calculated from the triplicate measurements (n = 3, *P < 0.05, Student’s t-test). (**C**) The e-scaffold decreases the viable cells of *A. baumannii* biofilms *in vitro* after 24 h of treatment. The data represent the means and standard errors of the means from four biological replicates (n = 4, *P ≤ 0.001, Student’s t-test). For these experiments, the e-scaffold was polarized at −600 mV_Ag/AgCl_ and the average current density was −60 μA/cm^2^.

Figure 2A shows sample images of green fluorescent protein (GFP)-expressing *A. baumannii* biofilms initially and after 24 h for both non-polarized (control) and polarized e-scaffolds. The control biofilms continued to grow into larger biofilm clusters over the 24-h period, whereas the biofilms disappeared after 24 h of exposure to the e-scaffold. These observations are consistent with our previous study demonstrating that biofilm is eliminated from 316L stainless steel surface in the presence of electrochemically generated H_2_O_2_^22^. After a 24-h application of the e-scaffold, the biofilm surface was reduced significantly, from 25.0 ± 2.0% to 7.0 ± 2.3% (*P* < 0.05, Student’s t-test), whereas the biofilm coverage increased to 34.0 ± 3.5% for the control biofilms (Fig. 2B). The colony-forming units (log (CFU/cm^2^) of e-scaffold-treated biofilms decreased to 4.35 ± 0.27; those of the control were 8.29 ± 0.05 (Fig. 2C).

### H_2_O_2_ generation by the e-scaffold is the dominant mechanism of action

The amount of H_2_O_2_ generated by the e-scaffold was calculated by integrating current over time as shown in Supplementary Fig. S1A and converting the integrated total current to moles of H_2_O_2_. This resulted in an estimate of ~45 mM H_2_O_2_ generated during a 24-h period.

**Figure 3:**
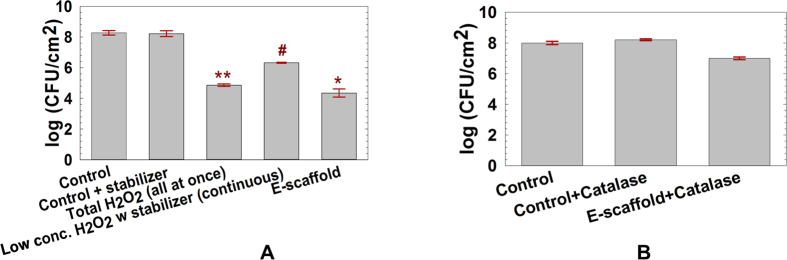
(**A**) H_2_O_2_ decreases the viable cells of *A. baumannii* biofilms *in vitro*. The error bars represent the standard errors of the means calculated from triplicate measurements. Symbols **, # and * denote a significant difference from the control (n ≥ 3, **P < 0.001; #*P* = 0.007; *P < 0.001, one-way ANOVA). (**B**) Exogenously added catalase decomposes H_2_O_2_ to oxygen and water and reduces biocidal activity. The error bars represent the standard errors of the means calculated from triplicate measurements. There was no significant difference from the control (*P* > 0.05, one-way ANOVA).

Interestingly, several researchers have reported that bacteria are eliminated near electrodes that have a negative DC polarity with a current density similar to that in our experiments (Table 1). Most investigators speculate that this antibacterial effect results from the negative electric current *per se*. Istanbullu *et al.*^22^ recently reported that H_2_O_2_ is generated by a polarized surface, and for the current study, we surmised that this is the mechanism responsible for the beneficial effects of an e-scaffold. As an independent test of this mechanism, we added exogenous H_2_O_2_ to *A. baumannii* biofilms. When 45 mM H_2_O_2_ was delivered in a single administration, there was a ~3-log reduction in *A. baumannii* CFU compared to the control without H_2_O_2_. This magnitude of reduction is similar to that of *in situ* biofilm reduction (Fig. 3A). However, the application of H_2_O_2_ at a concentration >100 μM is not practical, as it is reported to be cytotoxic for mammalian cells^37^,^38^,^39^. Continuous delivery at lower concentrations over longer periods of time reportedly removes biofilm without damaging mammalian cells^22^,^30^,^31^. When an equivalent concentration of H_2_O_2_ was delivered continuously over the course of 24 h, we observed only a ~2-log reduction in bacterial counts (Fig. 3B). While this latter experiment was expected to work as well as the e-scaffold, we surmise that the difference occurred because the e-scaffold provides a better distribution of H_2_O_2_ by electrotaxis and thus achieves greater biofilm reduction. The two stabilizers used in these experiments (sodium sulfate and manganese phosphate) had no independent effect on the biofilms (Fig. 3A).

We estimated that 0.01 mg/ml catalase was needed for complete decomposition of the H_2_O_2_ that was generated by the e-scaffold (Supplementary Fig. S1B). When a 5-fold greater concentration of catalase was applied to the biofilms, the e-scaffold only produced a ~1-log reduction in the number of viable cells (Fig. 3B), consistent with H_2_O_2_ being the principal bactericidal mechanism. The catalase itself had no significant effect on biofilm (Fig. 3B). Because the e-scaffold reduced the number of viable cells by ~4 log (Fig. 3A) and the addition of catalase blocked all but a ~1-log reduction (Fig. 3B), we concluded that the biocidal activity is due to electrochemically generated H_2_O_2_. The estimated 25% difference could be due to electrostatic or electrophoretic effects of negative electric current^40^,^41^ (Fig. 3). Alternatively, it is possible that H_2_O_2_ was not eliminated by the catalase enzyme rapidly enough and there was some loss of viable cells.

### The e-scaffold eliminates biofilm from infected porcine explants

**Figure 4:**
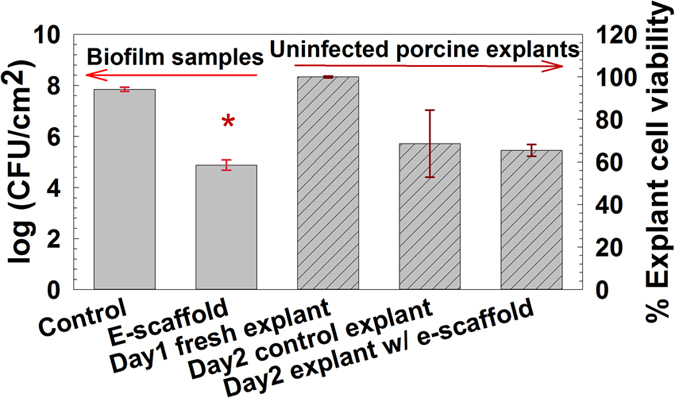
The e-scaffold decreases the number of viable cells of *A. baumannii* biofilms on infected porcine explants without affecting explant cell viability. Dark gray solid bars represent means from five independent *A. baumannii* biofilms. The error bars represent the standard errors of the means from five biological replicates (n = 5, *P < 0.001, Student’s t-test). For these experiments the e-scaffold was polarized at −600 mV_Ag/AgCl_ and the average current density was −56 μA/cm^2^. The gray patterned bars represent the percent viability of cells in the fresh (t = 0 h), control (untreated, t = 24 h) and e-scaffold treated (t = 24 h) uninfected porcine explants. The data are means from nine three porcine explant each with triplicate measurements, and the error bars represent standard errors of the means calculated from triplicate measurements. No significant difference between control and e-scaffold treated samples was observed (*P* = 0.85, one-way ANOVA).

The efficacy of H_2_O_2_ generated from the e-scaffold was tested against infected porcine explants. We observed a ~3-log reduction in colony-forming units (log (CFU/cm^2^) for e-scaffold treated biofilms (Fig. 4). We also found that 95% of the tissue cells in the e-scaffold treated uninfected explants remained viable compared to the untreated control, as measured using a viability stain (n = 3, *P* = 0.85, one-way ANOVA). Blinded histological assessments showed that there was no significant damage to the underlying tissue given exposure to the potentiated e-scaffold (Supplementary, Fig. S2 and Fig. S3).

Collectively these results demonstrate that the e-scaffold can reduce a biofilm community by four orders of magnitude by generating reactive H_2_O_2_ without apparent damage to the underlying tissue. Others have reported no tissue damage from the direct application of similar concentrations of H_2_O_2_^10^,^30^,^42^.

This work confirmed that the polarized e-scaffold successfully eliminates biofilms and that the electrochemical generation of H_2_O_2_ is the dominant mechanism of action. This e-scaffold design requires oxygen to diffuse to the carbon fiber surfaces^22^. If needed, to increase H_2_O_2_ production and concentration the e-scaffold could be exposed to an atmosphere enriched with oxygen^43^,^44^,^45^. In practice, the electrolyte medium could be replaced to prevent drying and to improve reaction rate while keeping the wound bed moist^9^,^16^,^29^.

## Conclusions

Considerable attention is being focused on alternative, antibiotic-free biofilm removal strategies. Electrochemically generated H_2_O_2_ near biofilm surfaces can eliminate the biofilm, but this requires continuous delivery at low concentrations in clinical settings. We have established the first step for an e-scaffold that continuously generates H_2_O_2_ at an applied potential of −600 mV_Ag/AgCl_ over the course of 24 h. With a maximum sustained concentration of ~25 μM H_2_O_2_ generated at any given time *in vitro*, the e-scaffold reduced the number of *A. baumannii* in biofilms by ~4 log. When it was applied to an infected porcine explant there was a ~3-log reduction in the biofilm community without detectable damage to the underlying mammalian tissues. Thus, the e-scaffold eliminates *A. baumannii* biofilm through a defined mechanism, and deserves further investigation as a wound treatment.

## Materials and Methods

### Electrochemical scaffold (e-scaffold)

The e-scaffold consisted of three electrodes. The working electrode held a negative polarity to reduce oxygen and generate H_2_O_2_. To complete the electrochemical cell, we used a counter electrode and a custom-made Ag/AgCl reference electrode. A custom-built e-scaffold was fabricated using carbon fabric (Panex 30 PW-06, Zoltek Companies Inc., St Louis, MO). The fabric was cut in a circular shape (6.42 cm^2^) to serve as the e-scaffold, and a smaller circular carbon fabric “patch” (2.14 cm^2^) was used as the counter electrode. The counter electrode was attached to the e-scaffold using a thin layer (~1 mm) of silicone rubber sealant (DAP Dynaflex 230 Premium Indoor/Outdoor Sealant, catalog #18357) that provided insulation between the electrodes while still allowing oxygen to diffuse to the bottom surface of the e-scaffold for H_2_O_2_ generation.

**Figure 5:**
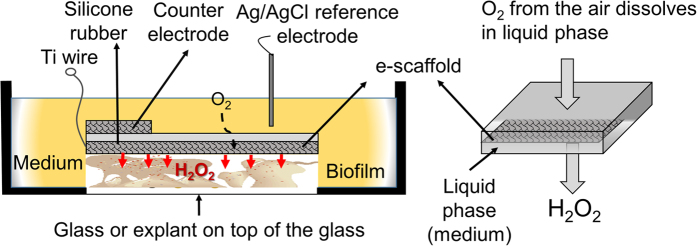
Schematic of experimental setup for treatment of biofilm exposed to e-scaffold with illustration of electrochemical H_2_O_2_ production. Electrodes are connected to a potentiostat (not shown in figure). Microscope glass coverslips and porcine explants were used as biofilm growth surfaces for *in vitro* and infected porcine explant experiments, respectively.

For controlled generation of H_2_O_2_, precise and accurate control of the potential of the e-scaffold is essential^22^. Therefore, the electric potential applied to the e-scaffold was controlled using a Gamry Series G 300™ potentiostat (Gamry Instruments, Warminster, PA, USA) against a saturated Ag/AgCl reference electrode^6^. Ti wires (0.025 Ti, Malin Co., Cleveland, OH, Lot #27567) were used to connect the electrode ends to the external cables leading to a potentiostat (Fig. 5). The connection resistance was consistently <2 Ω. The e-scaffold was overlaid either onto biofilms grown *in vitro* on glass surface or onto infected or uninfected porcine explants (Fig. 5). This configuration allowed the ventral surface of the e-scaffold to be exposed directly to biofilms.

### Quantifying H_2_O_2_ production from the e-scaffold

H_2_O_2_ production was quantified by coupling both linear sweep voltammetry and constant polarization of the e-scaffold with the direct measurement of H_2_O_2_ using a H_2_O_2_ microelectrode^22^. Initially, the microelectrode tip (<20 μm) was positioned above the e-scaffold (~1000 μm) using a precision linear actuator (PI M-230.10S, Physik Instrumente, Auburn, MA, USA) controlled using custom software (LabVIEW, National Instruments, Austin, TX, USA). The microelectrode tip and the e-scaffold surface were located using a stereomicroscope (Zeiss Stemi 2000). The microelectrode tip was then moved down to within ~50 μm of the e-scaffold surface (Supplementary, Fig. S4). At this position linear sweep voltammetry was initiated from +400 mV_Ag/AgCl_ to −800 mV_Ag/AgCl_ at 10 mV/s. The onset of H_2_O_2_ production was measured from this voltammetry, and −600 mV_Ag/AgCl_ was selected as the optimum potential to produce H_2_O_2_ near the surface. Following linear sweep voltammetry, the e-scaffold was polarized to −600 mV_Ag/AgCl_ and the current was allowed to reach a steady value. Starting at 1000 μm from the e-scaffold surface, the microelectrode tip was stepped down in 5-μm increments towards the e-scaffold surface. After each increment the H_2_O_2_ concentration was measured to develop a depth-resolved concentration profile. The accumulation of H_2_O_2_ at the e-scaffold surface and the penetration distance into the bulk were measured using these depth profiles. The depth profile of H_2_O_2_ for a non-polarized e-scaffold surface was similarly measured as a control. The profiles were measured on one side since the other side was insulated by a layer of inert silicone rubber sealant and no reaction happens on that surface.

### Growing *in vitro* biofilms

To test the efficacy of e-scaffold at destroying existing biofilm, we used *Acinetobacter baumannii* biofilms in this study. An overnight culture of *A. baumannii* (ATCC #BAA-1605) was grown in full-strength Luria Broth (LB) medium (Sigma-Aldrich, catalog #L3522) and was resuspended in 5% LB medium (OD_600_ ≈ 0.5). For imaging experiments, GFP expressing *A. baumannii* (ATCC #17978) was used and LB medium was supplemented with ampicillin (100 μg/mL; Sigma-Aldrich, catalog #A5354-10ML). Sterile glass bottom petri dishes (MatTek Corporation, catalog #P35G-1.5-20-C) were used to grow and image the biofilms (Supplementary, Fig. S5A). After 2 h of initial attachment, the bacterial suspension was removed and the biofilms were washed twice to remove planktonic cells and then refreshed with 5% LB medium. Biofilms were allowed to develop for 24 h. Before the e-scaffolds were applied, the bulk liquid was refreshed.

### Application of e-scaffold to *in vitro* biofilms

E-scaffolds were sterilized by autoclaving (121 °C, 15 min) and were saturated with sterilized liquid medium. Then the bulk liquid above the existing *A. baumannii* biofilms were removed carefully and the e-scaffolds were placed onto it. Fresh medium was added to the system. The scaffolds were then polarized at –600 mV_Ag/AgCl_ for 24 h, after which biofilms were processed for the quantification of viable cells by scraping the e-scaffold and biofilms from the glass surfaces of the petri dish into 5 ml of LB medium (1 g/L). These suspensions were centrifuged, the resulting cell pellet was resuspended in 1 ml of LB medium (1 g/L), and serial dilutions were prepared. Colony-forming units (CFU) were counted using a drop-plate count method^46^. Biofilms exposed to non-polarized e-scaffolds were used as a control.

### Biofilm imaging and analysis

An inverted epifluorescence microscope (Nikon Eclipse Ti-S inverted microscope) with a Nikon DS-Qi1Mc camera and a CFI Plan Fluor ELWD 40X objective (N.A. 0.60, W.D. 3.72.7 mm) was used to image the cells. Biofilms were imaged before exposure to the e-scaffold (t = 0 h) and after 24 h of exposure. To remove any planktonic cells, biofilms were washed twice and refreshed with 5% LB medium supplemented with ampicillin prior to imaging. Image Structure Analyzer (ISA) was used to calculate surface coverage by biomass from the digitized biofilm images automatically^6^. At least ten discrete images were taken each time to obtain statistically representative data^47^. Average values were calculated for these ten images. The average values of three biological replicates were used to calculate the means and standard errors. We used surface coverage, which is the ratio of the area of the biomass to the total area of the image, as the main indicator of biofilm structure. The higher the percentage surface coverage, the higher the coverage of the glass surface by biofilms.

### External H_2_O_2_ addition

To test whether H_2_O_2_ by itself can remove biofilms *in vitro* and from infected porcine explants, we added exogenous H_2_O_2_ (VWR, Catalog #RC3819-16, adjusted to a concentration similar to that produced by the e-scaffold) to *A. baumannii* biofilms. The total amount of H_2_O_2_ generated from the e-scaffold was estimated by charge balance calculations from equation (1)^6^ and the integration of current vs. time data observed from the potentiostat (Supplementary, Fig. S1A). First, we challenged the biofilms with the total calculated amount of H_2_O_2_ (45 mM) in a single administration. Then, in separate experiments, we added H_2_O_2_ continuously (similar to the e-scaffold) to biofilms at an average of 2 mM/h for 24 h. This allowed us to simulate the total amount of H_2_O_2_ produced over time by the e-scaffold. To minimize the rapid oxidation of H_2_O_2_ we added stabilizers (0.005% sodium sulfate and 0.003% manganese phosphate) to the solution. The stabilizers were also included in a separate control treatment.

### External catalase addition

Catalase decomposes H_2_O_2_ and thereby blocks its biocidal activity^48^,^49^. We added catalase (Sigma-Aldrich, catalog #C1345) to *A. baumannii* biofilm and measured its protection against H_2_O_2_ produced by the e-scaffold. Prior to the addition, the H_2_O_2_ decomposition rate per unit of catalase was determined from H_2_O_2_ microelectrode measurements. The total amount of catalase required per min for the complete decomposition of H_2_O_2_ was calculated based on the rate of H_2_O_2_ generation by the e-scaffold (Supplementary, Fig. S1B). To ensure complete H_2_O_2_ decomposition, catalase was added in excess of the calculated value (5×
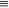
0.05 mg/ml). In a separate experiment we tested the ability of this amount of catalase to inhibit biofilms.

### Biofilm-infected porcine explants

The e-scaffold was tested against biofilm-infected porcine explants. We followed previously published protocols to prepare infected explants^50^,^51^. Ear tissues were harvested from domestic pigs (obtained from C&L Lockers, Moscow, ID, USA), immediately cooled to 4 °C and kept for less than an hour at this temperature before being processed at the laboratory. No purpose-bred animals were used for these experiments. After the tissues were cleaned with 70% ethanol and the hair was removed using an electric razor, skin was excised with a scalpel. For the intact epidermis model the excised skin was sectioned at a thickness of approximately 500 μm, using Padgett’s dermatome, and punched into 12-mm-diameter discs, excluding skin with visible structural changes (scratches, erosion or scars). For the partial cutaneous wound model, mid-dermal sheets with a thickness of 500 μm were harvested^52^. Skin punches with the dermal side down were used to seed polycarbonate transwell inserts (Greiner Bio-One North America, Inc., catalog #657641) with a 0.4-μm pore size membrane separating each explant from the outer well prefilled with 2 ml of cell nutrient medium. These were maintained at 37 °C and 95% humidity in a 5% CO_2_ environment. The nutrient medium consisted of serum-free Dulbecco’s Modified Eagles Medium (DMEM) (Thermo Scientific, catalog #SH3024301) supplemented with L-glutamine (0.584 g/L), ampicillin (50 μg/ml) and antifungal amphotericin B (0.4 μg/ml). Biofilms were initiated by adding 5 μl of overnight culture of *A. baumannii* (ATCC #17978, OD_600_ ≈ 0.5) to the center of each explant surface. After 4 days the biofilm-infected porcine explants were ready to use.

### Application of the e-scaffold to infected porcine explants

E-scaffolds were prepared as described above and overlaid onto *A. baumannii* biofilm-infected porcine explants. The inner well with the explant and e-scaffold was filled with 4 ml of sterile PBS as electrolyte (Supplementary, Fig. S6). Similar to the *in vitro* experiment, e-scaffold surface exposed to biofilm was polarized at −600 mV_Ag/AgCl_. Biofilms exposed to non-polarized e-scaffolds were used as a control. After 24 h of polarization, the e-scaffolds as well as the explants with biofilms from both polarized and control wells were collected and processed for serial dilution and bacterial cell counts as described above.

### Cytotoxicity test of the e-scaffold on porcine explants

The cell viability in the uninfected porcine explants with induced wounds was quantified to test whether the polarized e-scaffold treatment damaged the tissues^50^. After application of the polarized e-scaffold for 24 h, the porcine explant cell viability was quantified using PrestoBlue cell viability reagent (Life Technologies, catalog #A-13261) with the standard protocol (Life Technologies). Briefly, explants were incubated in 300 μL of 10% PrestoBlue (in DMEM) for 3 h at 37 °C in an environment with 95% humidity and 5% CO_2_. The absorbance of the medium was then measured at 570 nm and 600 nm. The percent reduction of PrestoBlue was calculated from this absorbance and the molar extinction coefficient of oxidized and reduced PrestoBlue. The extent of PrestoBlue reduction calculated for explants exposed to polarized e-scaffolds was compared to that of the control, i.e. explants with non-polarized e-scaffolds. A normalized viability score of 100% was given to the explant showing the highest percent reduction of PrestoBlue for this control.

### Histopathology

Where indicated replicate tissue explants were fixed in 10% neutral buffered formalin and 5-μm-thick sections were prepared and stained with matoxylin and eosin by the Washington Animal Disease Diagnostic Laboratory. The resulting light micrographs were subjected to a treatment-blind evaluation by a board-certified veterinary anatomic pathologist.

### Statistical Analysis

Unless indicated otherwise, all experiments were performed using three independent replicates. The average results were expressed as mean ± standard deviation and analyzed using one-way ANOVA and Student’s t-test to identify any significant difference between samples with and without e-scaffold application. Statistical analyses were performed using SigmaPlot (version 12.5).

## Additional Information

**How to cite this article**: Sultana, S. T. *et al.* Electrochemical scaffold generates localized, low concentration of hydrogen peroxide that inhibits bacterial pathogens and biofilms. *Sci. Rep.* **5**, 14908; doi: 10.1038/srep14908 (2015).

## Supplementary Material

Supplementary Information

## Figures and Tables

**Table 1:** Examples of publications on direct current electrical stimulation for wound treatment.

Bacterial strain/wound type	Dressing current/applied Potential	Polarity of the electrodeplaced on the wound	Suggested mechanism	Applicationtime	Study type	Reference
Extended spectrum β-lactamase bacteria, multidrug-resistant bacteria, and methicillin-resistant *Staphylococcus aureus (isolated from complex wounds in a patient’s limb)*	Procellera Ag-Zn composite bioelectric dressing (generating 0.3 to 0.9 V)	Positive (compared to negatively charged bacteria)	Antimicrobial components Ag and Zn generated near a positive pole caused either bacteriostatic or bactericidal activity depending on the bacterial strain under study	24 h48 h	*In vitro*	53
*S. aureus*	PPY/Chitosan film 25 μA/cm^2^	Negative (compared to a secondary gold electrode)	DC enhanced autolysis of bacteria in biofilm	24 h (4 h/day)	*In vitro*	40
*Pseudomonas aeruginosa (isolated from a patient’s wound)*	Stainless steel electrodes (1.5, 3.5, 5.5, 10 V DC)	Positive (compared to a secondary stainless steel electrode)	A toxic electrochemical product (not identified) formed around an electrode with a positive pole caused bacterial growth inhibition.	19 h	*In vitro*	20
		Negative (compared to a secondary stainless steel electrode)	Maximum inhibition at 3.5 V, with current creating a bacteriostatic property or bactericidal effect	19 h		
*P. aeruginosa (infected skin wound model)*	Copper mesh electrode 10–52 μA/cm^2^	Negative (compared to a secondary copper mesh electrode)	Bacteriostatic or bactericidal effect of electric current	Total 14 weeks with sampling every 24 h (72 h treatment was the most stable and successful)	*In vivo* rabbit wound model	15
Mixed bacteria (not specified) (*infected wounds of various types*)	Stainless steel mesh or carbon electrode materials 30–110 μA/cm^2^	Negative (compared to secondary stainless steel or carbon electrode)	Electrostatic and/or electrochemical influence was involved.	2 h (treatment twice daily for 5 days a week)	Random clinical trial on hospital patients	14
*Pseudomonas* and/or *Proteus* species *(skin ulcers)*	Copper mesh electrode 8–31 μA/cm^2^	Negative	Uncertain	6 h (three times a day, each for 2 h)	Random clinical trial on hospital patients	16
*Staphylococci* spp., *Pseudomonas* spp. etc. *(Venous leg ulcers)*	Surgical steel gauze 75–100 μA (current density unknown)	Negative	Negative electric current influencing oxygen reduction and local attraction of special cations	30–40 days (dressing changed weekly)	Clinical trials	13

## References

[b1] Dijkshoorn L., Nemec A. & Seifert H. An increasing threat in hospitals: multidrug-resistant Acinetobacter baumannii. Nat Rev Micro 5, 939–951, 10.1038/nrmicro1789 (2007). 18007677

[b2] Costerton J. W. & Stewart P. S. Battling biofilms - The war is against bacterial colonies that cause some of the most tenacious infections known. The weapon is knowledge of the enemy’s communication system. Sci Am 285, 74–81 (2001). 11432197 10.1038/scientificamerican0701-74

[b3] Del Pozo J. L., Rouse M. S. & Patel R. Bioelectric effect and bacterial biofilms. A systematic review. Int J Artif Organs 31, 786–795 (2008). 18924090 10.1177/039139880803100906 PMC3910516

[b4] Freebairn D. *et al.* Electrical methods of controlling bacterial adhesion and biofilm on device surfaces. Expert Rev Med Dev 10, 85–103, 10.1586/erd.12.70 (2013). 23278226

[b5] Kirketerp-Moller K. *et al.* Distribution, organization, and ecology of bacteria in chronic wounds. J Clin Microb 46, 2717–2722, 10.1128/jcm.00501-08 (2008). PMC2519454 18508940

[b6] Lewandowski Z. & Beyenal H. in Fundamentals of Biofilm Research, 2^nd^ edn (Taylor & Francis, 2013).

[b7] Hampton S. Malodorous fungating wounds: how dressings alleviate symptoms. Brit J Community Nurs 13, S31-passim (2008). 10.12968/bjcn.2008.13.Sup3.29470 18773764

[b8] Ip M., Lui S. L., Poon V. K. M., Lung I. & Burd A. Antimicrobial activities of silver dressings: an *in vitro* comparison. J Med Microb 55, 59–63, 10.1099/jmm.0.46124-0 (2006). 16388031

[b9] Sweeney I. R., Miraftab M. & Collyer G. A critical review of modern and emerging absorbent dressings used to treat exuding wounds. Int Wound J 9, 601–612, 10.1111/j.1742-481X.2011.00923.x (2012). 22248337 PMC7950558

[b10] Banerjee J. *et al.* Improvement of Human Keratinocyte Migration by a Redox Active Bioelectric Dressing. Plos One 9, 10.1371/journal.pone.0089239 (2014). PMC3940438 24595050

[b11] Kanokpanont S., Damrongsakkul S., Ratanavaraporn J. & Aramwit P. An innovative bi-layered wound dressing made of silk and gelatin for accelerated wound healing. Int J Pharm 436, 141–153, 10.1016/j.ijpharm.2012.06.046 (2012). 22771972

[b12] Tkachenko O. & Karas J. A. Standardizing an *in vitro* procedure for the evaluation of the antimicrobial activity of wound dressings and the assessment of three wound dressings. J Antimicrob Chemother 67, 1697–1700, 10.1093/jac/dks110 (2012). 22514261

[b13] Assimacopoulos D. Low intensity negative electric current in the treatment of ulcers of the leg due to chronic venous insufficiency. Preliminary report of three cases. Am J Surg 115, 683–687, 10.1016/0002-9610(68)90101-3 (1968). 5300518

[b14] Carley P. J. & Wainapel S. F. Electrotherapy for acceleration of wound-healing-low-intensity direct-current. Arch Phys Med Rehabil 66, 443–446 (1985). 3893385

[b15] Rowley B. A., McKenna J. M., Chase G. R. & Wolcott L. E. The influence of electrical current on an infecting microorganism in wounds. Ann N Y Acad of Sci 238, 543–55, 10.1111/j.1749-6632.1974.tb26820.x (1974). 4216282

[b16] Wolcott L. E., Wheeler P. C., Hardwicke H. M. & Rowley B. A. Accelerated healing of skin ulcers by electro therapy preliminary clinical results. South Med J 62, 795–801 (1969). 5306004 10.1097/00007611-196907000-00008

[b17] Ojingwa J. C. & Isseroff R. R. Electrical stimulation of wound healing. J Invest Dermatol 121, 1–12 (2003). 12839557 10.1046/j.1523-1747.2003.12454.x

[b18] Isseroff R. R. & Dahle S. E. Electrical Stimulation Therapy and Wound Healing: Where Are We Now? Adv Wound Care 1, 238–243, 10.1089/wound.2011.0351 (2012). PMC3839020 24527312

[b19] Thakral G. *et al.* Electrical stimulation to accelerate wound healing. Diabet Foot Ankle 4, 10.3402/dfa.v4i0.22081 (2013). PMC3776323 24049559

[b20] Maadi H. *et al.* Effect of alternating and direct currents on Pseudomonas aeruginosa growth *in vitro*. Afr J Biotechnol 9, 6373–6379 (2010).

[b21] Del Pozo J. L. *et al.* Effect of Electrical Current on the Activities of Antimicrobial Agents against Pseudomonas aeruginosa, Staphylococcus aureus, and Staphylococcus epidermidis Biofilms. Antimicrob Agents Chemother 53, 35–40, 10.1128/aac.00237-08 (2009). 18725436 PMC2612137

[b22] Istanbullu O., Babauta J., Nguyen H. D. & Beyenal H. Electrochemical biofilm control: mechanism of action. Biofouling 28, 769–778, 10.1080/08927014.2012.707651 (2012). 22827804 PMC4247835

[b23] Hong S. H. *et al.* Effect of electric currents on bacterial detachment and inactivation. Biotechnol Bioeng 100, 379–386, 10.1002/bit.21760 (2008). 18080346

[b24] Busalmen J. P. & De Sanchez S. R. Adhesion of Pseudomonas fluorescens (ATCC 17552) to nonpolarized and polarized thin films of gold. Appl Environ Microb 67, 3188–3194, 10.1128/aem.67.7.3188-3194.2001 (2001). PMC92999 11425740

[b25] Bard A. J., Parsons R. & Jordan J. in Standard Potentials in Aqueous Solution (Taylor & Francis, 2001).

[b26] Vetter K. J. in Electrochemical Kinetics: Theoretical and experimental aspects (Academic Press, 1967).

[b27] Drosou A., Falabella A. & Kirsner R. S. Antiseptics on Wounds: An Area of Controversy. Wounds 15 (2003).

[b28] Guo S. & Dipietro L. A. Factors affecting wound healing. J Dent Res 89, 219–229, 10.1177/0022034509359125 (2010). 20139336 PMC2903966

[b29] Zhao G. *et al.* Biofilms and Inflammation in Chronic Wounds. Adv Wound Care 2, 389–399 (2013). 10.1089/wound.2012.0381 PMC3763221 24527355

[b30] Loo A. E. K. *et al.* Effects of Hydrogen Peroxide on Wound Healing in Mice in Relation to Oxidative Damage. PLoS ONE 7, e49215, 10.1371/journal.pone.0049215 (2012). 23152875 PMC3496701

[b31] Linley E., Denyer S. P., McDonnell G., Simons C. & Maillard J. Y. Use of hydrogen peroxide as a biocide: new consideration of its mechanisms of biocidal action. J Antimicrob Chemother 67, 1589–1596, 10.1093/jac/dks129 (2012). 22532463

[b32] Babauta J. T., Nguyen H. D., Istanbullu O. & Beyenal H. Microscale Gradients of Oxygen, Hydrogen Peroxide, and pH in Freshwater Cathodic Biofilms. Chemsuschem 6, 1252–1261, 10.1002/cssc.201300019 (2013). 23766295 PMC4247834

[b33] Flick B A; *Argentum Medical, LLC. Conductive wound dressings and methods of use. United States patent US 8,449,514. 2013 May 28*.

[b34] Langenhove L. V. in Smart Textiles for Medicine and Healthcare: Materials, Systems and Applications Vol. 63 (Woodhead publishing, 2007).

[b35] Yang X. M., Beyenal H., Harkin G. & Lewandowski Z. Evaluation of biofilm image thresholding methods. Water Res 35, 1149–1158, 10.1016/s0043-1354(00)00361-4 (2001). 11268835

[b36] Del Pozo J. L. *et al.* The Electricidal Effect Is Active in an Experimental Model of Staphylococcus epidermidis Chronic Foreign Body Osteomyelitis. Antimicrob Agents Chemother 53, 4064–4068, 10.1128/aac.00432-09 (2009). 19651912 PMC2764171

[b37] Burdon R. H. Superoxide and hydrogen peroxide in relation to mammalian cell proliferation. Free Radic Biol Med 18, 775–794, 10.1016/0891-5849(94)00198-S (1995). 7750801

[b38] Kiyoshima T. *et al.* Oxidative stress caused by a low concentration of hydrogen peroxide induces senescence-like changes in mouse gingival fibroblasts. Int J Mol Med 30, 1007–1012, 10.3892/ijmm.2012.1102 (2012). 22922974 PMC3573718

[b39] Wijeratne S. S., Cuppett S. L. & Schlegel V. Hydrogen peroxide induced oxidative stress damage and antioxidant enzyme response in Caco-2 human colon cells. J Agric Food Chem 53, 8768–8774, 10.1021/jf0512003 (2005). 16248583

[b40] Zhang J., Neoh K. G., Hu X. & Kang E.-T. Mechanistic insights into response of Staphylococcus aureus to bioelectric effect on polypyrrole/chitosan film. Biomaterials 35, 7690–7698, 10.1016/j.biomaterials.2014.05.069 (2014). 24934644

[b41] van der Borden A. J., van der Mei H. C. & Busscher H. Electric block current induced detachment from surgical stainless steel and decreased viability of Staphylococcus epidermidis. Biomaterials 26, 6731–6735, 10.1016/j.biomaterials.2004.04.052 (2005). 15979141

[b42] Harding A. C., Gil J., Valdes J., Solis M. & Davis S. C. Efficacy of a Bio-electric Dressing in Healing Deep, Partial-thickness Wounds Using a Porcine Model. Ostomy Wound Manag 58, 50–55 (2012). 22933701

[b43] Kranke P., Bennett M. H., Roeckl‐Wiedmann I. & Debus S. Hyperbaric oxygen therapy for chronic wounds. Cochrane Database Syst Rev 2, CD004123 (2004). 15106239 10.1002/14651858.CD004123.pub2

[b44] Wright J. *Hyperbaric oxygen therapy for wound healing. Technical report*. (2001) Avaialable at: worldwidewounds.com (Accesseed: 23^rd^ January 2015).

[b45] Doctor N., Pandya S. & Supe A. Hyperbaric oxygen therapy in diabetic foot. Journal of postgraduate medicine 38, 112 (1992). 1303408

[b46] Chen C. Y., Nace G. W. & Irwin P. L. A 6 × 6 drop plate method for simultaneous colony counting and MPN enumeration of Campylobacter jejuni, Listeria monocytogenes, and Escherichia coli. J Microbiol Methods 55, 475–479, 10.1016/s0167-7012(03)00194-5 (2003). 14529971

[b47] Ica T. *et al.* Characterization of mono- and mixed-culture Campylobacter jejuni biofilms. Appl Environ microbioly 78, 1033–1038, 10.1128/AEM.07364-11 (2012). PMC3273011 22179238

[b48] Liu X. F., Roe F., Jesaitis A. & Lewandowski Z. Resistance of biofilms to the catalase inhibitor 3-amino-1,2,4-triazole. Biotechnol Bioeng 59, 156–162, 10.1002/(sici)1097-0290(19980720)59:2<156::aid-bit3>3.0.co;2-g (1998). 10099326

[b49] Stewart P. S. *et al.* Effect of catalase on hydrogen peroxide penetration into Pseudomonas aeruginosa biofilms. Appl Environ microbioly 66, 836–838, 10.1128/aem.66.2.836-838.2000 (2000). PMC91906 10653761

[b50] Lone A. G. *et al.* Staphylococcus aureus induces hypoxia and cellular damage in porcine dermal explants. Infect immun, IAI-03075 (2015). 10.1128/IAI.03075-14 PMC4432762 25847960

[b51] Burd A. *et al.* A comparative study of the cytotoxicity of silver-based dressings in monolayer cell, tissue explant, and animal models. Wound Repair Regen 15, 94–104, 10.1111/j.1524-475X.2006.00190.x (2007). 17244325

[b52] Jacques C. *et al.* Percutaneous absorption and metabolism of [14C]-ethoxycoumarin in a pig ear skin model. Toxicol In Vitro 24, 1426–1434, 10.1016/j.tiv.2010.04.006 (2010). 20417268

[b53] Kim H. *et al.* Antibacterial efficacy testing of a bioelectric wound dressing against clinical wound pathogens. Open Microbiol J 8, 15–21, 10.2174/1874285801408010015 (2014). 24627730 PMC3950956

