# Supplementary material for: Electrochemical scaffold generates localized, low concentration of hydrogen peroxide that inhibits bacterial pathogens and biofilms
Source: Sci Rep. 2015 Oct 14;5:14908. doi: 10.1038/srep14908 (PMC4604468; doi:10.1038/srep14908)
Supplement: Supplementary Information [file srep14908-s1.pdf]

Supporting Information for

Electrochemical scaffold generates localized, low concentration of hydrogen peroxide that inhibits bacterial pathogens and biofilms

Sujala T Sultana[1], Erhan Atci[1], Jerome T Babauta[1], Azeza Mohamed Falghoush[2], Kevin R. Snekvik[2,3], Douglas R. Call[2,4] and Haluk Beyenal[1*]

[1]School of Chemical Engineering & Bioengineering, Washington State University, Pullman 99163, WA.
[2]Department of Veterinary Microbiology and Pathology, Washington State University, Pullman 99163, WA.
[3]Washington Animal Disease Diagnostic Laboratory, Washington State University, Pullman 99163, WA.
[4]Paul G. Allen School for Global Animal Health, Washington State University, Pullman 99163, WA.
*beyenal@wsu.edu

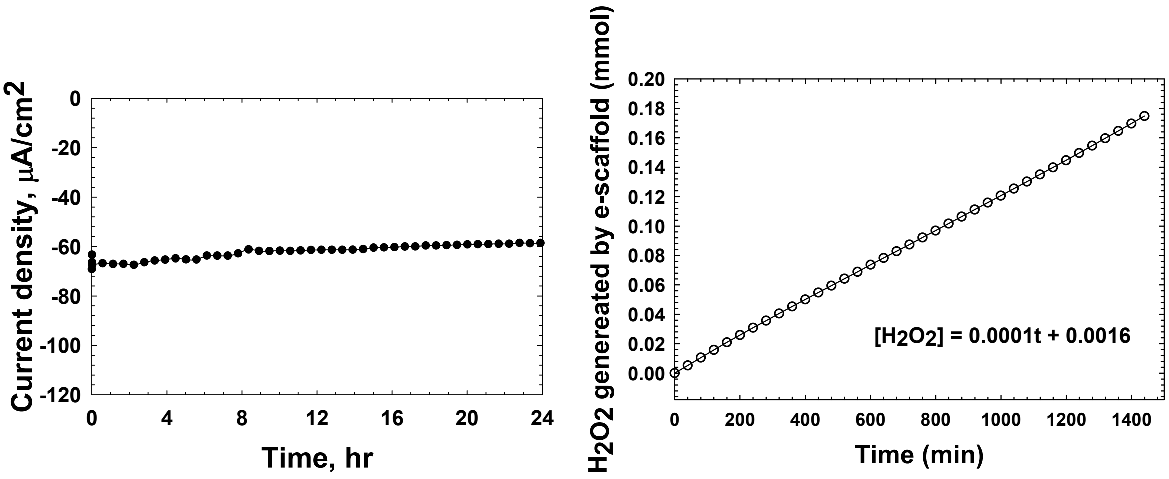

A **B**

Fig. S 1. A) Current variation in polarized e-scaffold over 24 h when polarized at -600 $mV_{Ag/AgCl}$. B) H_2O_2 generation rate over time calculated from the current data in A. The slope of the equation indicates the H_2O_2 generation rate was approximately 0.1 μmol/min (~0.03 mM/min in our system).

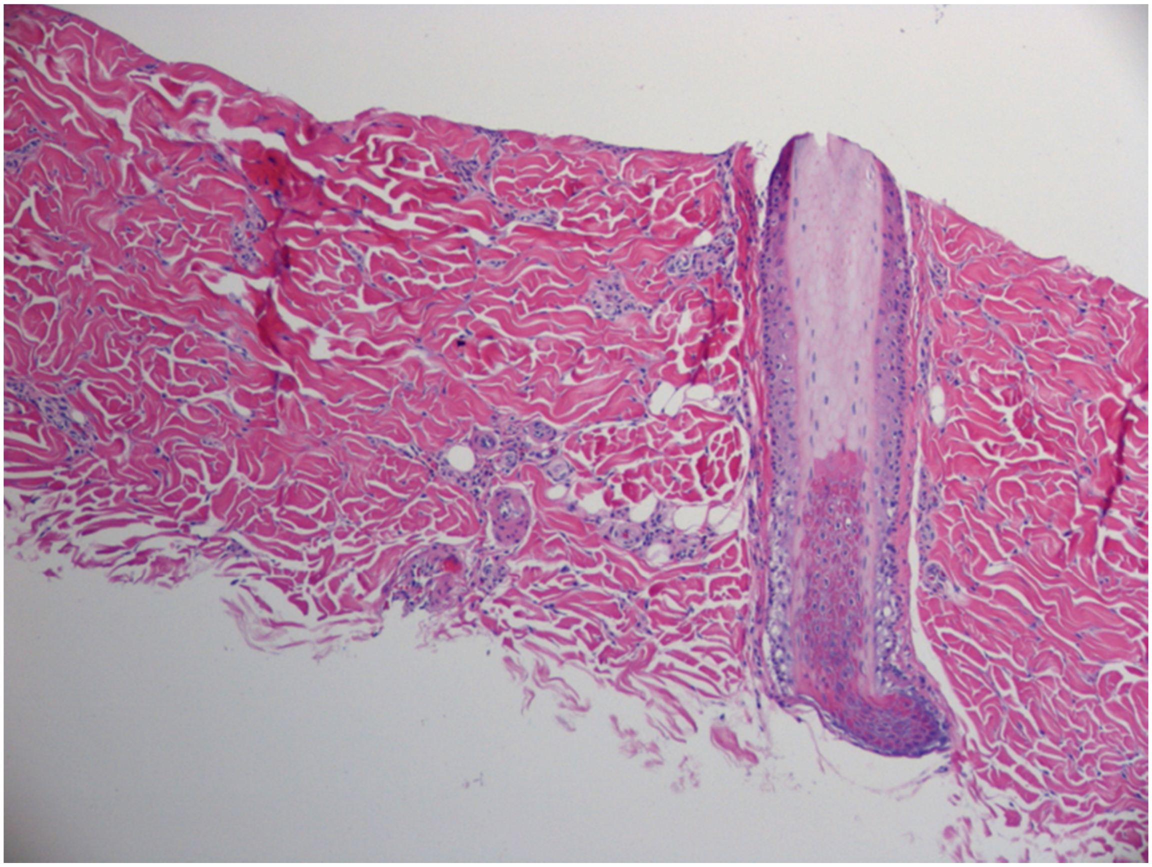

Fig. S 2. Micrograph image of 2-day-old uninfected porcine explant with no exposure to e-scaffold. A normal hair follicle is present in the dermis. Superficial dermal vessels are cuffed by low numbers of lymphocytes, plasma cells, eosinophils and macrophages.

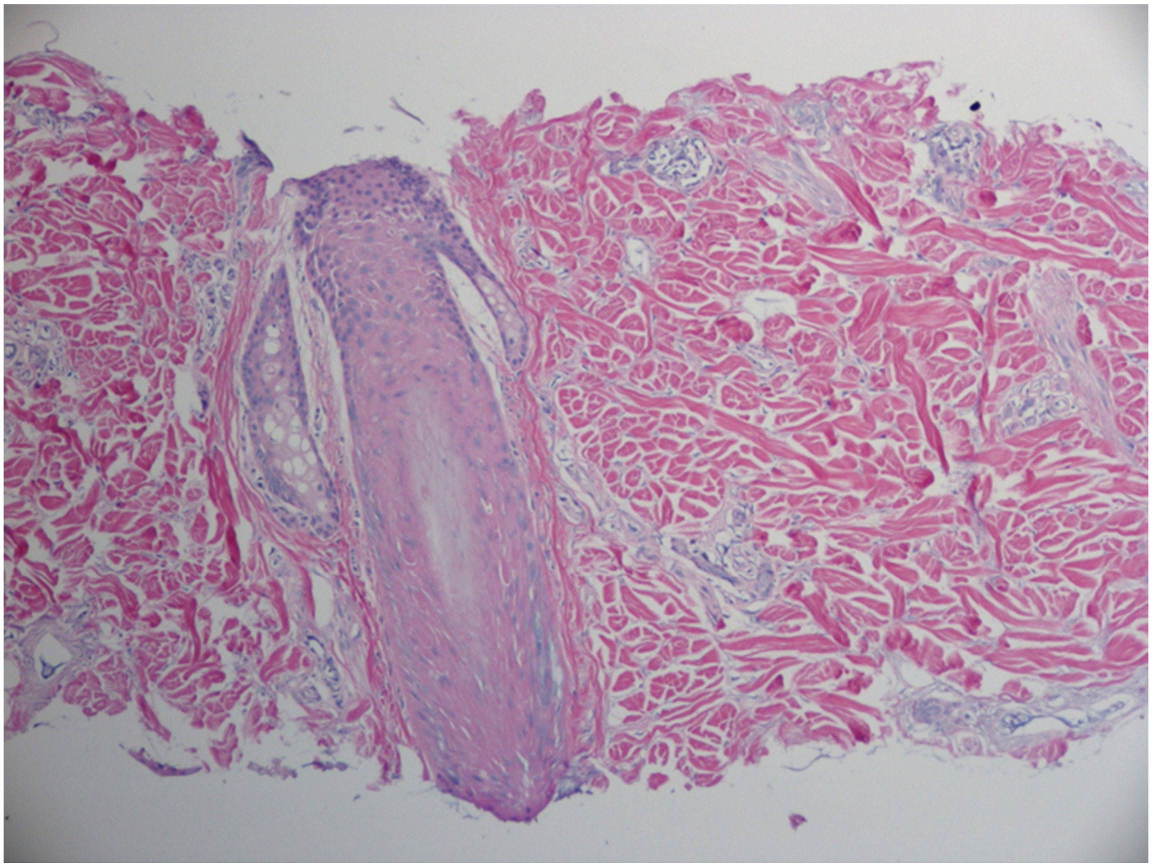

Fig. S 3. Micrograph image of 2-day-old uninfected porcine explant. A potentiated e-scaffold was overlaid onto the tissue for the last 24 h before tissues were removed for histological analysis. No gross changes are evident relative to Fig. S 2. (above), although there is some minor loss of differential staining with minimal loss of cellular detail.

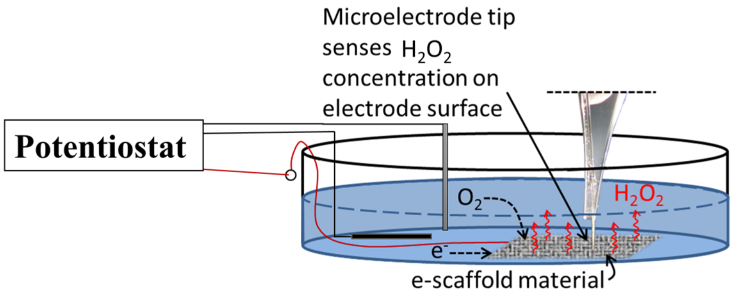

Fig. S 4. Schematic of the setup for quantifying H_2O_2 production at the polarized e-scaffold surface using a H_2O_2 microelectrode. The onset potential of H_2O_2 production and the concentration of H_2O_2 at the e-scaffold surface were measured.

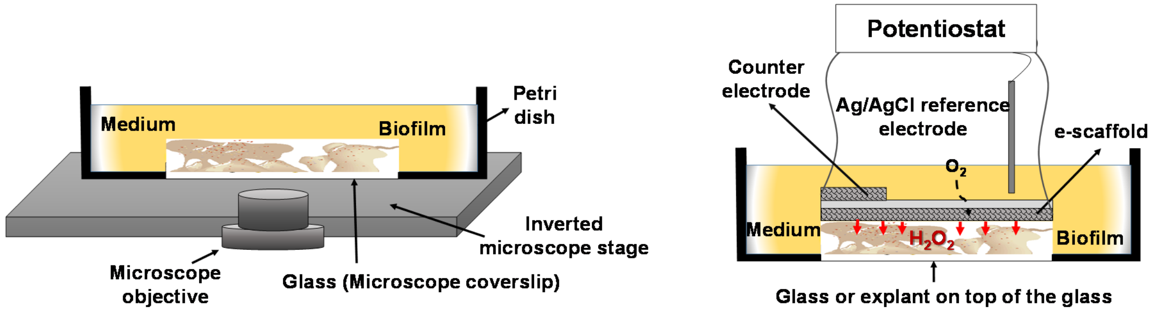

A **B**

Fig. S 5. A) Schematic of the experimental setup for growing and imaging *in vitro* biofilm. B) Schematic of the experimental setup for treating biofilm with an e-scaffold. A potential of -600 $mV_{Ag/AgCl}$ was applied using a potentiostat.

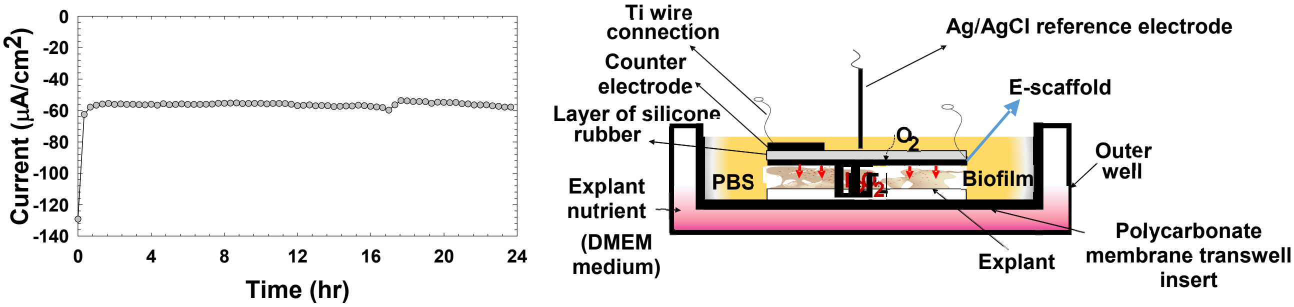

A **B**

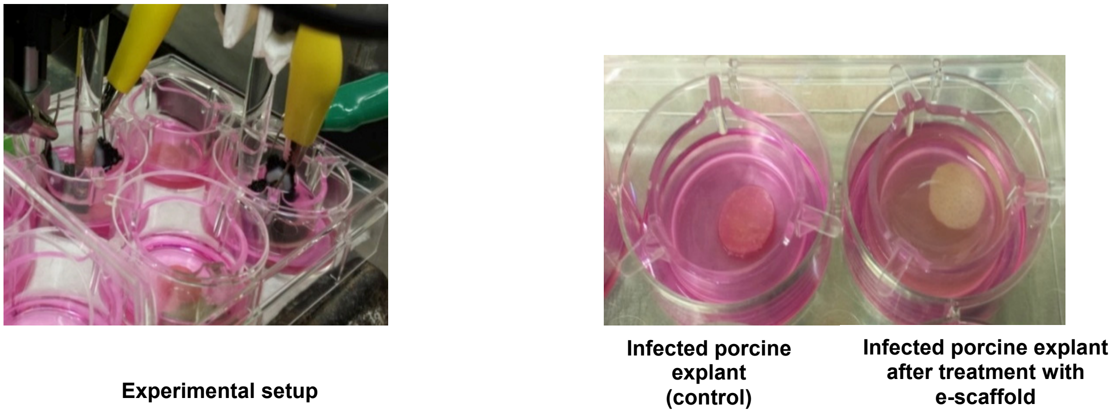

C **D**

Fig. S 6. A) Current variation in e-scaffold over 24 h when polarized at -600 $mV_{Ag/AgCl}$.
B) Schematic of experimental setup for e-scaffold overlaid on infected porcine explants.
C) Photograph of experimental setup for e-scaffold overlaid on biofilm on explants.
D) E-scaffold treated and untreated infected porcine explants. The biofilm infection in the control explant is visible; whereas the e-scaffold treated explant looks cleaner.
